# Enhancing hydrodynamic efficiency in Autonomous Underwater Vehicles (AUVs) utilizing adjoint method and proper orthogonal decomposition

**DOI:** 10.1371/journal.pone.0319321

**Published:** 2025-03-31

**Authors:** Shorob Alam Bhuiyan, Md. Jahid Hasan, Md. Araful Hoque

**Affiliations:** 1 Department of Mechanical, Aerospace, and Nuclear Engineering, Rensselaer Polytechnic Institute, Troy, New York, United States of America; 2 Department of Mechanical and Production Engineering (MPE), Islamic University of Technology (IUT), Board Bazar, Gazipur, Bangladesh; 3 Department of Mechanical and Production Engineering (MPE), Ahsanullah University of Science and Technology, Dhaka, Bangladesh; Embry-Riddle Aeronautical University, UNITED STATES OF AMERICA

## Abstract

In recent years, there has been a significant need for high-performing and efficient Autonomous Underwater Vehicles (AUVs). This is primarily because of their use in offshore mineral exploitation and oceanographic research. While there have been notable breakthroughs in applying the adjoint technique to optimize air and land vehicles, there is still a deficiency in optimizing AUVs using the adjoint method. The present research explores how to improve the hydrodynamic efficiency of an AUV using the gradient-based adjoint technique and Proper Orthogonal Decomposition (POD). This study especially tries to minimize drag forces on the entire AUV by exclusively employing the adjoint approach on the AUV’s wing. The simulation was conducted using computational fluid dynamics methodology utilizing the Reynolds-averaged Navier–Stokes (RANS) model, with velocities ranging from 0.5 m/s to 2 m/s. Numerical computations demonstrated significant reductions in drag force, with the most advantageous improvements obtained when the wing geometry was altered by 9%. More precisely, the optimization resulted in a 9% drop in drag force at a speed of 1 m/s, going from 98.91 N to 90.17 N. By traveling at a speed of 2 m/s, a significant 17% reduction in drag force was achieved, reducing it from 386.34 N to 320.90 N. This signifies a substantial improvement of 20.25% in power consumption. The POD technique was employed to determine the dominant modes in the flow field, resulting in improved simulations and a better comprehension of flow patterns.

## 1. Introduction

Optimizing the aerodynamic and hydrodynamic characteristics is a vital goal in various industries, such as automotive, aeronautics, and maritime engineering. Engineers strive for exceptional performance in these areas, and the incorporation of Computational Fluid Dynamics (CFD) into design methodologies has significantly increased the ability to forecast and improve the interaction between fluid dynamics and structures. This development has resulted in significant improvements in performance and efficiency.

CFD analysis entails the representation of issues using appropriate mathematical equations and accurate boundary conditions. Afterward, numerical methods are used to address these problems and predict precise consequences of the system, such as drag, downforce, and flow characteristics [1]. Continuous improvements in processing power and numerical analytical methods drive the increasing importance of CFD in design. CFD applications significantly reduce the time and financial resources needed for the development of innovative designs [2]. The efficacy of CFD simulations in enhancing the aerodynamic efficiency of race cars is exemplified by Hetawal et al. [3] through comparative analyses conducted on three distinct automotive categories. Installing a front wing can decrease the coefficient of drag of a typical sports car from 0.85 to 0.70, for instance. A study conducted by Shen Ai et al. [4] and Buscariolo et al. [5] revealed that mirrors on a vehicle had a substantial influence, obscuring around 3% of the frontal area. In addition, Ali et al. discovered that modifying the mirrors’ design might reduce the drag coefficient of cars by as much as 4.9%. In a separate investigation carried out by Qin et al. [6], the aerodynamic characteristics of a standard car design called DrivAer were analyzed using CFD. Researchers analyzed different types of mesh and solver algorithms and concluded that using a hybrid RANS-LES (Large Eddy Simulation) approach is a beneficial compromise for obtaining precise and cost-efficient simulations of aerodynamic forces. Furthermore, it was shown that the drag coefficient and lift coefficient were hardly affected by the distance downstream. The most significant deviations, amounting to 0.7% and 4.5% respectively, were seen when comparing various distances. The selection of the correct solver in CFD is essential for precise prediction and analysis of aerodynamic performance, as evidenced by the research undertaken by A. Patil and J. Navrátil [7]. The researchers have found that employing unstable RANS simulations produces superior outcomes compared to steady RANS simulations while studying the aerodynamic performance of NASA’s X-57 aircraft wing under different configurations. Efficiently manipulating and improving the flow of liquids through numerical methods is essential for both academic research and practical applications in industry.

A crucial factor in the design of vehicles that operate in fluid environments, such as aircraft or AUVs, is the meticulous consideration of aerodynamic or hydrodynamic design. Since water is denser than air, effective drag-reduction techniques are crucial for decreasing power consumption and improving vehicle efficiency. Over the last few decades, notable advancements have been made in designing and controlling autonomous underwater vehicle systems [8]. A study conducted by Hadipour and Rad [9] examined the impact of raising the backward sweep angle of the horizontal tail and hydrofoil of a conventional submarine. The results showed that increasing the horizontal tail sweep angle by 50 degrees resulted in a 6% reduction in drag compared to the condition with zero backward sweep angle. In addition, when the hydrofoil backward sweeps, the angle is increased to 60 degrees, and there is a 14% reduction in drag. Mahmoudian’s dissertation [10] and Linklater’s thesis [11] both investigate control systems for underwater vehicles, contributing to the existing body of study in this field. Mahmoudian’s research showcases a control system designed for underwater gliders, with a particular focus on achieving energy-efficient trajectories using a combination of stable wing-level and turning motions. Linklater’s research focuses on analyzing the dynamics, stability, and control of a two-part towed underwater vehicle. The study reveals that implementing a Proportional Integral Derivative (PID) controller greatly improves the vehicle’s pitch and roll stability, effectively keeping the tilt angles under ±0.5 degrees even in changing conditions. These works demonstrate progress in motion control for underwater vehicles, specifically in optimizing path efficiency and ensuring stability. Mission-specific underwater autonomous craft now commonly employ unorthodox designs. Such a study was done by Miller et al. [12], where they utilized toroidal hull form for an AUV and achieved 13% drag reduction. Bioinspired design has been employed to enhance the performance of AUVs, as demonstrated in research conducted by Zixuan Li [13], Li et al. [14], and Gan et al. [15]. Zixuan Li worked on the development of an AUV that takes inspiration from squids. The length of the vehicle’s head is decided to balance viscous drag and pressure drag while minimizing the overall drag in the AUV design. In addition, a disk is placed at the front of the AUV to minimize fluid contact, resulting in a reduction of viscous drag and an overall decrease in drag by 5.34%. On the other hand, another study conducted by Zhao et al. [16] utilized core microstructures inspired by pufferfish spines in suboff models. Their findings indicate that placing these microstructures at the front of the submarine leads to a proportional increase in the drag reduction rate as the distance from the highest point increases. This demonstrates an inverse relationship between the spacing of the microstructures and the rate of drag reduction.

The adjoint method is a highly effective optimization technique that refines design parameters by decreasing drag forces, which is crucial for achieving operational efficiency and environmental sustainability. The efficacy of the adjoint approach in enhancing the aerodynamic efficiency of automobiles. Studies conducted by Jiarong Wu, Yiding Wen [17], Othmer [18] and Maksimov et al. [19], Wang et al. [20] and Hu et al. [21] have explored the use of adjoint optimization to enhance vehicle performance. Their research revealed that optimizing the body shape of vehicles can improve the streamlining of fluid flow, resulting in increased speed and reduced drag. Hucho [22] states that the upper body of a vehicle is responsible for generating 45% of the aerodynamic drag force. The underbody and wheel housing contribute the majority of the aerodynamic force, accounting for around 55%. Furthermore, Ahsan et al. [23] demonstrate that the side-view mirrors account for approximately 4.8% of the overall drag coefficient. The studies conducted by Magazoni et al. [24] revealed that slight alterations to the surface form of the view mirror can have a significant impact on axial force. The rearview mirror’s structure is particularly prone to decreasing the axial force, which in turn reduces the drag coefficient. Therefore, an optimized surface is created by utilizing the morphing approach and analyzing the outcomes of the sensitivity. The overall force falls by 9.98 N, which corresponds to a drag coefficient of 0.008. In conditions such as high-speed driving and turning, the aerodynamic forces and moments on a race car are primarily determined by the driving speed and the shape of the car body [25]. Research done by Kalinowski and Szczepanik [26] found the downforce-to-drag ratio equal to 4.48, which is twice the size of the non-optimized front wing, enhanced the performance of the race car. This improvement directly impacts the vehicle’s velocity and maneuverability. Hoque et al. [27] conducted a study where they analyzed a formula student vehicle. The adjoint solver was utilized to optimize the car’s geometry, particularly the nose, with respect to other design variables. When evaluated at a Reynolds number of 10.8 ×  10^5^, the streamlined nose demonstrated a 27% reduction in drag and a 27.09% drop in fuel consumption compared to the typical design. Moreover, the OpenFOAM combined approach was employed to optimize the aerodynamics of an AUDI Q5. The results were then compared to CFD calculations and wind tunnel testing, allowing for a comprehensive analysis of the problems and identification of best practices [28].

There are a range of optimization methods applied across various domains, each with its strengths and limitations. Table 1 summarizes these studies, highlighting the methods used, their applications, their strength, and limitations. This comparison underscores the novelty of combining adjoint optimization with Proper Orthogonal Decomposition (POD) for improving the hydrodynamic performance of Autonomous Underwater Vehicles (AUVs).

**Table 1:** Comparison of optimization methods in literature and their relevance to the current study.

Author	Optimization Method used	Application	Strengths	Limitations
Antunes and Azevedo [29]	Genetic Algorithm	Airfoil	This method is excellent at exploring large and complex solution spaces without requiring gradient information	A complicated integrated system, often slow to converge, especially for high-dimensional or highly nonlinear problems
Akter et al. [30]	High-fidelity panel method	Airfoil	It provides accurate solutions for potential flow problems	The method is intensive and less effective for capturing viscous effects or highly turbulent flows
He et al. [31]	Particle Swarm Optimization	High-speed train	These algorithms are simple to implement, require minimal parameter tuning, and are effective for solving nonlinear and multi-modal optimization problems	They can suffer from slow convergence in high-dimensional search spaces
Zohora et al. [32]	Iterative design process	Microchannel heat sink	This method allows for continuous refinement and improvement of designs based on feedback, ensuring solutions are tailored to specific requirements	Time-consuming and resource-intensive

Adjoint optimization is also utilized in other domains such as in railway engineering, and high-speed trains are considered one of the most efficient means of transportation nowadays. A study done by Paniagua et al. [33] found that optimizing the nose of the Inter-City Express (ICE) train by adjoint method reduced drag by 7.2%. The adjoin approach is also employed for glider optimization, in a study conducted by Amalia et al. [34], an incidence angle of 2 degrees was applied to the wing, followed by the optimization process. The lift coefficient (C_L_) baseline is 0.81, and the drag coefficient (C_D_) value is 0.0271. The wing that was optimized attained a maximum C_L_ of 1.02 and a C_D_ of 0.0362. A comparable study on AUV utilizing the adjoint approach was conducted, employing OpenFOAM v2206 with adjointOptimisationFoam to enhance the nose and tail sections of the AUV. Attaining a 3.25% reduction in drag [35].

The principle of Navier equations is a mathematical design used to study the movement of viscous fluids. It is beneficial for analyzing phenomena such as the formation of drag vortices and other fluid flow characteristics. Combining the discrete adjoint method of optimization with governing equations can create an aerodynamic body geometry that maximizes fuel efficiency and minimizes drag. This technique involves iterative processes that adjust geometry parameters to achieve the least amount of drag while meeting all other requirements [36–38]. This study investigates the optimization of naval architecture, notably focusing on the design of a winged AUV. It takes inspiration from advancements in optimizing terrestrial and aerial vehicles. Streamlined underwater vehicles (SUVs), also known as Autonomous underwater vehicles (AUVs) in certain literature, are used for oceanographic research, offshore mineral exploitation, fisheries acoustics, and pipeline surveillance [39]. In the early stages of SUV-shape optimization, the total resistance of the underwater vehicle was analyzed by separating it into friction and shape resistance. These two components were then evaluated independently using empirical methods [40–42]. A study conducted by Chen and Liu [43] yielded significant findings in optimizing SUV form by utilizing an adaptive sampling technique. A multi-objective particle swarm optimization–crowding distance approach is used to address multi-objective optimization issues. The SUV with the most significant number of curvature parameters produced the most optimal set of solutions for multiple objectives and showed a 6% improvement compared to the preliminary SUV.

Computers have enhanced our ability to replicate complex systems and gather and scrutinize vast quantities of data. Subsequently, one could analyze hundreds of millions of data points to get a small number of interesting final quantities. In order to address this problem, one successful approach is to reduce order designing techniques. Among the different approaches for deriving preliminary flow structures, POD has been extensively employed (Berkooz et al. [44]; Holmes et al. [45]; Hu et al. [46]). This study presents a clear demonstration of POD, which was introduced to turbulence research by Lumley. POD is a robust and sophisticated technique for analyzing data, with the goal of producing concise descriptions of complex processes using fewer dimensions. Zhang et al. [47] conducted a study on the flow of cavitation wakes around a cylinder. They gained valuable knowledge on the intricate characteristics of both cavitating and non-cavitating wake flows. The study emphasized the influence of cavitation on vortex shapes and the dissipation of energy. Under non-cavitating conditions, 80 modes account for 91.67% of the total energy. During cavitation, the dominant mode accounts for 65.62% of the total, while the first 15 modes together account for 64.24%. Caraballo et al. [48] conducted a study examining the application of POD on Supersonic Axisymmetric Jet. The study discovered that a jet enlarged to Mach 1.4 is optimal, and it evaluated the usefulness of the snapshot POD approach in capturing the dynamics of coherent structures in a high-speed jet.

Based on the literature, it can be seen that adjoint shape optimization is a well-established method in various disciplines; however, its true potential in Autonomous Underwater Vehicles (AUVs) remains unexplored. This study addresses this gap by combining adjoint optimization with Proper Orthogonal Decomposition (POD) methods and introduces a unique approach to optimizing the wing geometry of an AUV designed by Olmos et al. [49]. The primary objective is to reduce drag forces on the AUV wing, which is a critical component for minimizing resistance and improving hydrodynamic efficiency. Lower drag forces lead to reduced power consumption, which is essential for enhancing operational performance. This research makes a novel contribution to naval engineering by bridging theoretical optimization frameworks with practical applications.

## 2. Problem Descriptions and Designing

**Fig 1:**
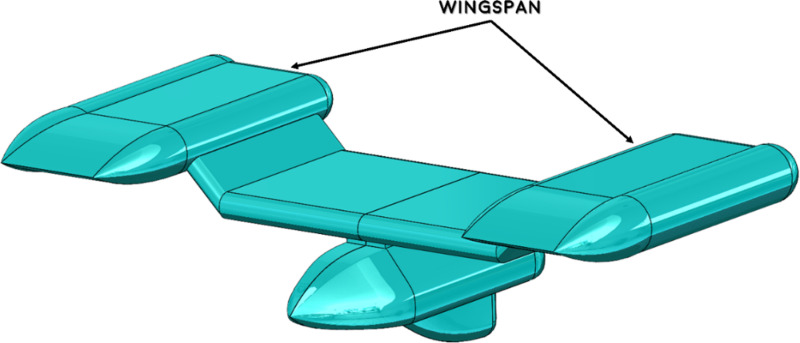
Computer-aided design of Autonomous Underwater Vehicle (AUV).

**Fig 2:**
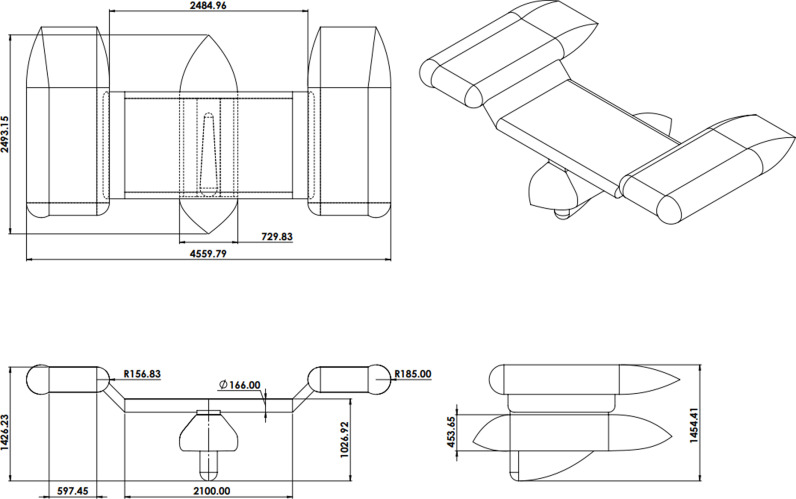
Autonomous Underwater Vehicle (AUV) dimensions (all dimensions are in mm).

This study aims to optimize the size of the wing on an autonomous winged vehicle (AUV). The AUV’s design is derived from the research conducted by Olmos et al. [49]. In this work, they developed an AUV and conducted an analysis of its underwater lift and drag. In addition, a design was created to accurately reproduce specific instances of the AUV. In this study to improve the hydrodynamic efficiency of the AUV, emphasis was made on optimizing the wing geometry, a crucial element in minimizing drag. Fig 1 illustrates the AUV, whereas Fig 2 provides a detailed visual representation of the AUV’s design specs. The primary emphasis is on the wing, which has a length of 2388.04 mm and a height of 370 mm. Fig 11 illustrates the wing design parameters in comparison to the enhanced wing design. The experiment utilized a velocity range spanning from 0.5 m/s to 2 m/s. Furthermore, a tetrahedral mesh was chosen due to its efficacy in representing the complicated geometry of the AUV, particularly the detailed characteristics of the wing and the adjacent flow zones. The computational domain was segmented into two primary regions: the near field, utilizing finer meshing to precisely capture essential flow characteristics such as wake regions, boundary layer interactions, and separation zones, and the far field, employing coarser elements to conserve computational resources while maintaining accuracy. An inflation layer was integrated into the mesh design to precisely depict the boundary layer. Additional information regarding the mesh configuration is available in Section 3.5 of this study.

**Fig 3:**
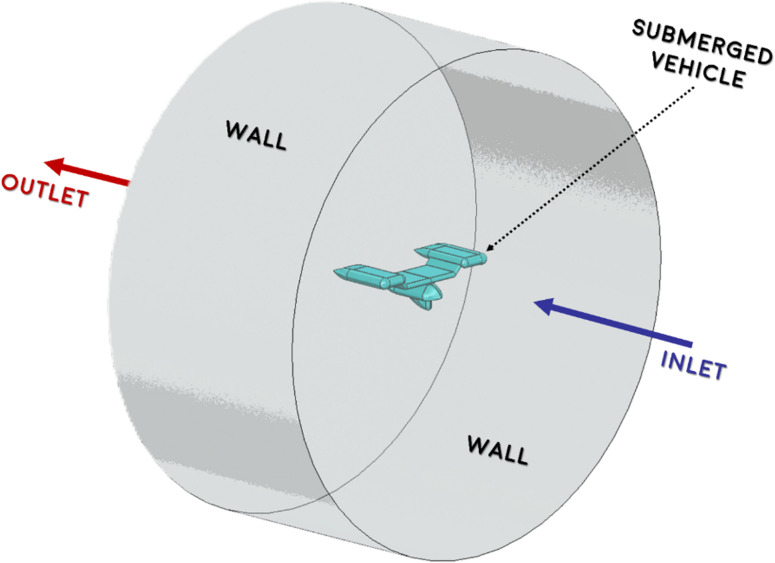
Submerged Vehicle within the Fluid Domain.

**Fig 4:**
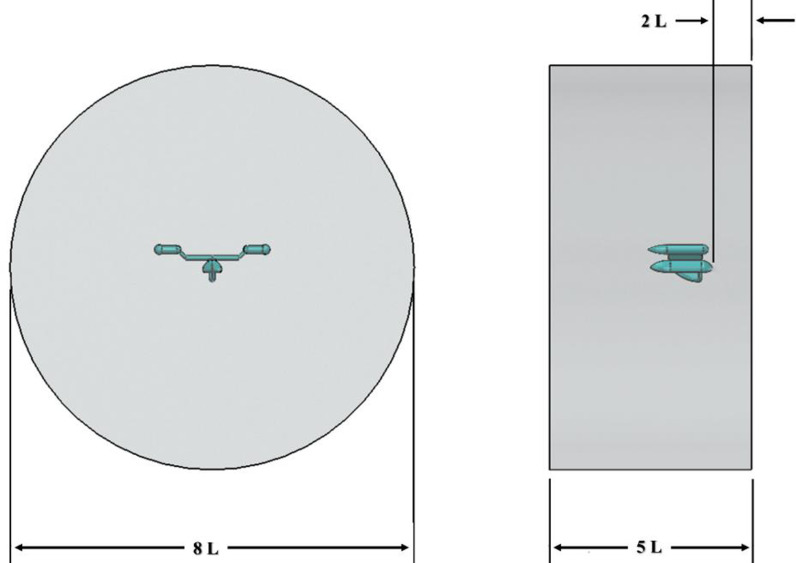
Dimensions of the fluid domain.

**Fig 5:**
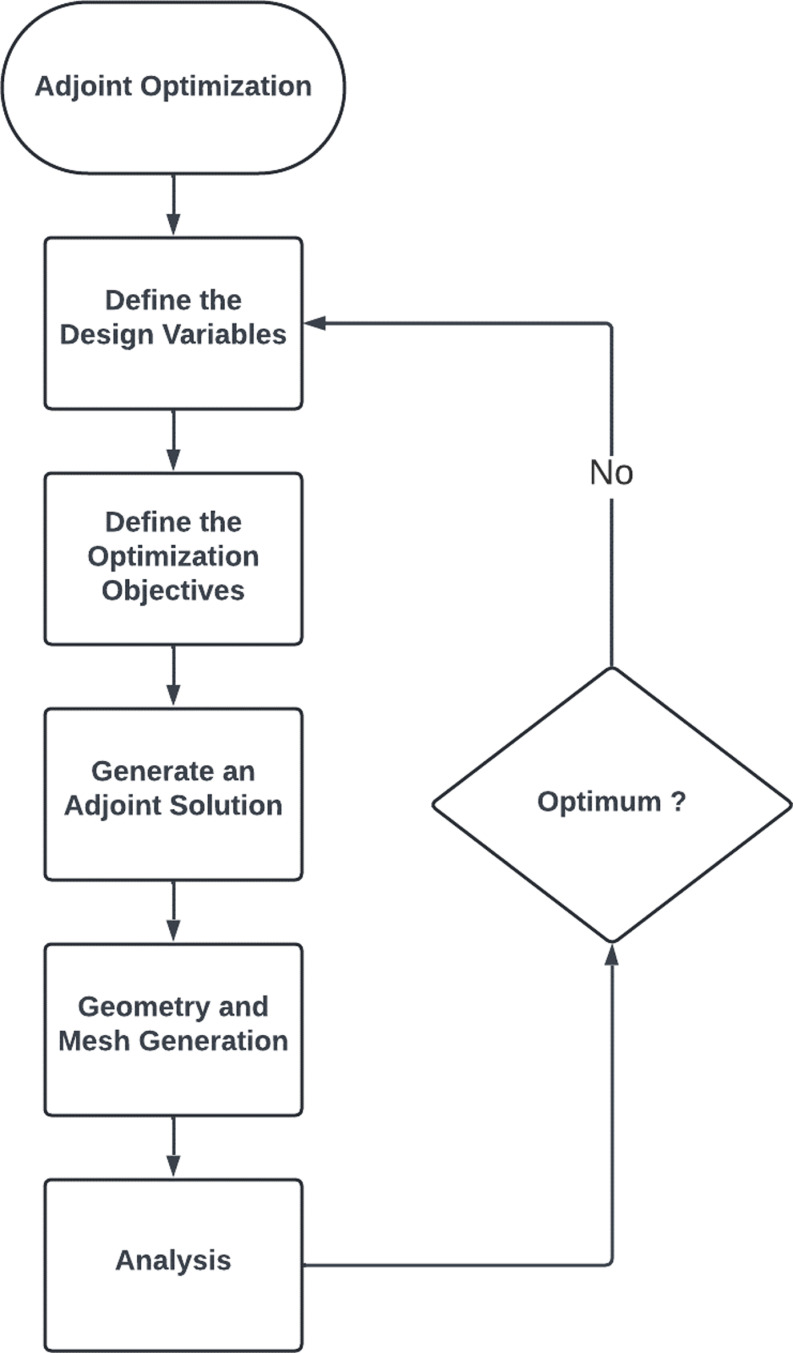
Adjoint optimization approach and steps.

**Fig 6:**
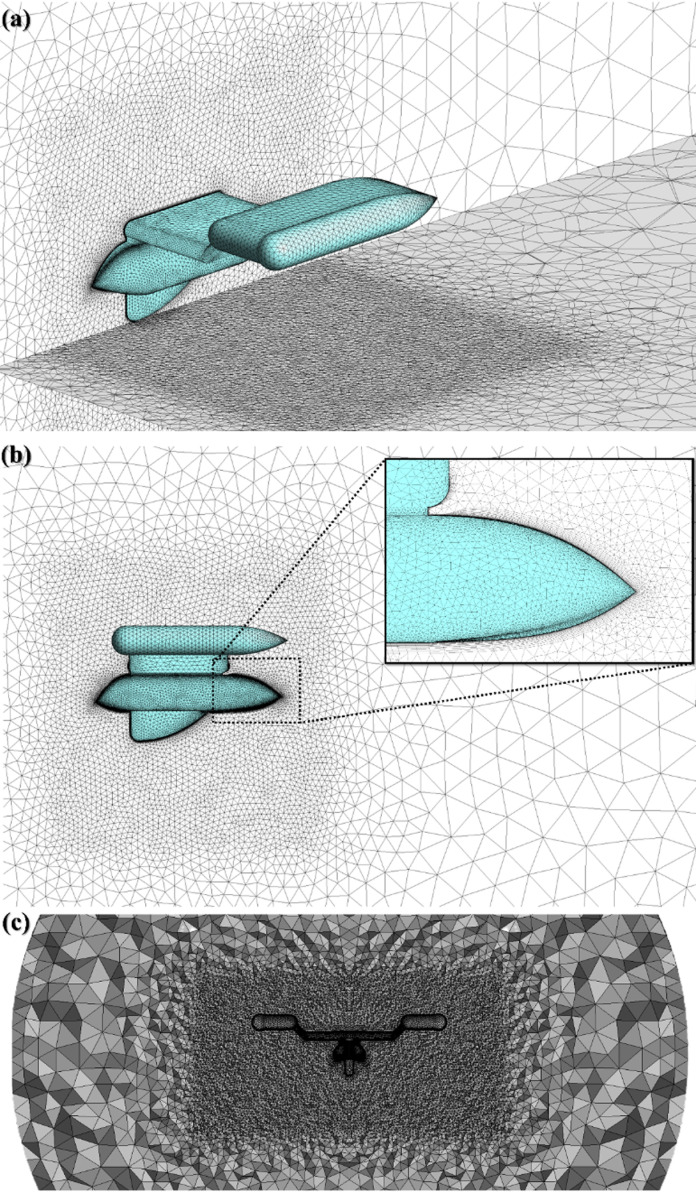
Mesh used in the system, a) Iso metric view; b) Side view; c) Front section view.

**Fig 7:**
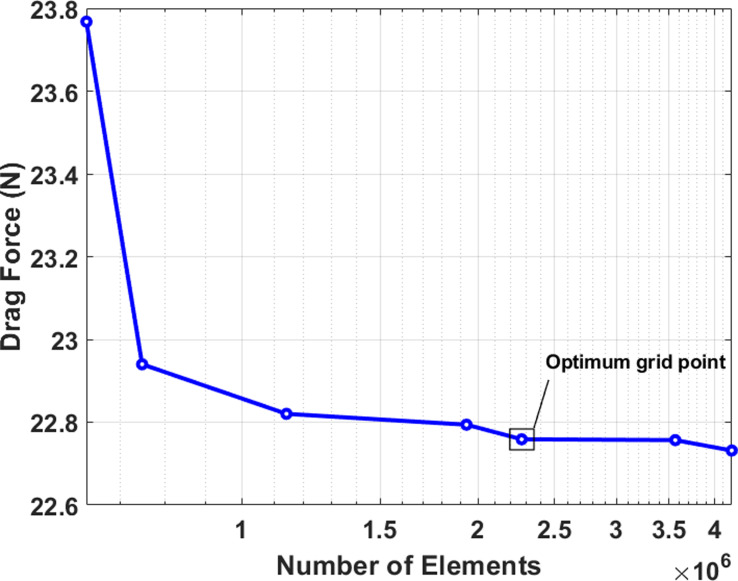
Grid independence test.

**Fig 8:**
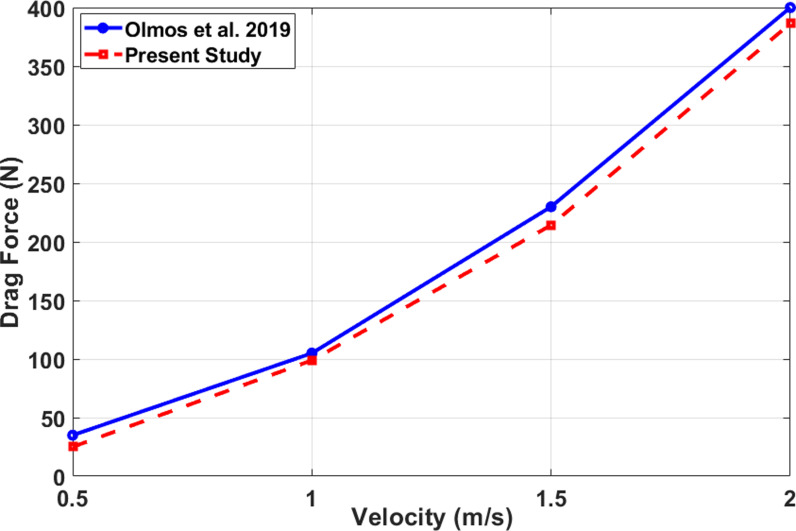
Evaluation of the data obtained by Olmos et al. [ 49] to the current study at different velocities.

**Fig 9:**
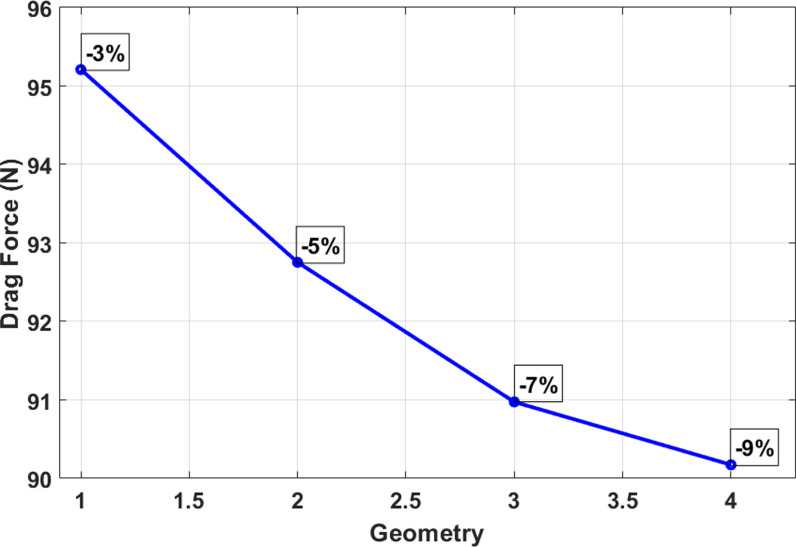
Effect of AUV wing percentage changes on drag reduction.

**Fig 10:**
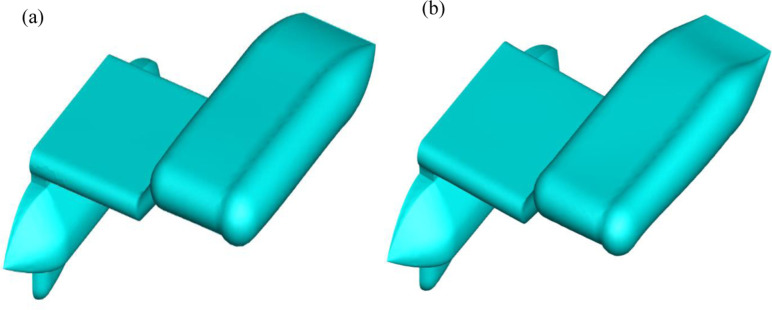
Iso metric view comparison of (a) Preliminary and (b) Optimized design.

In this work, the adjoint approach was applied to a specific component of the AUV, specifically the wing, due to its significant contribution to the AUV’s movement in water. The Adjoint approach was employed to optimize the wing’s design to minimize the drag experienced when fully immersed. The Adjoint solver identifies regions with elevated pressure and adjusts the corresponding portion of the geometry to decrease the pressure, leading to shape modifications that minimize pressure drag. The evaluation procedure entails analyzing and interpreting crucial characteristics, including drag coefficient, pressure distribution, and patterns in the wake flow. Once the simulations are finished, the collected data is meticulously examined to ascertain pertinent flow characteristics and performance indicators.

To conduct a more in-depth analysis of the research, the technique of proper orthogonal decomposition, also known as reduction order designing, was incorporated. To simplify the intricacy of computational designs, POD is a robust method employed to detect the most energetic modes in the flow field. For several reasons, this research subjected a steady-state simulation to POD analysis. Firstly, steady-state simulations are less computationally demanding than transient simulations, enabling a more efficient analysis. Furthermore, the flow patterns observed in steady-state simulations exhibit stability and remain constant throughout time, facilitating the identification and analysis of the prevailing modes. Finally, employing a steady-state simulation enables a concentrated examination of crucial flow patterns that have a substantial influence on the performance of the AUV, without the added intricacy of transient impacts. There are many advantages of employing POD in this research. POD aids in minimizing the data needed to represent the flow field, resulting in more efficient simulations. It offers a more lucid understanding of the prevailing flow patterns and their interplay. POD ensures precise capture of critical flow properties by prioritizing the most significant modes. Moreover, POD enhances the optimization procedure by emphasizing the crucial factors that impact performance, assisting in the creation of more effective wing configurations for the AUV. Integrating POD into the adjoint technique improves the overall efficiency of the optimization process, resulting in a more precise and effective design of the AUV wing.

## 3. Numerical study

### 3.1. Computational domain

A cylindrical fluid domain was generated to simulate the airflow around the AUV, as shown in Fig 3. The boundary requirements depicted in Fig 3 can be succinctly described as a symmetry condition imposed on the “center plane” and a free slip wall condition enforced on the outer curving surface. Concerning both the entry and exit conditions, the velocity in Cartesian coordinates was uniformly allocated to both the intake and outflow. The submerged design features a vertical gap between the body and the inlet that is 0.7 L, and a horizontal gap between the inlet and outlet that is 3.5 L. The minimum distance to the “Wall” is 4 L units as shown in Fig 4. The vehicle is positioned to ensure that the flow is fully developed before it encounters the front surface.

### 3.2 Mathematical equations

The Navier-Stokes equation is essential for accurately representing the fluid dynamics of an AUV that is underwater. The equation governing the preservation of mass, momentum, and energy in fluid movement is referred to as the conservation equation.

#### The continuity equation.

The continuity equation symbolizes the concept of conserving mass, for incompressible flow the continuity equation can be mathematically formulated as follows:


∇⋅u=0 (1)

The symbol denotes the molecular weight (*ρ*) of the fluid, which is quantified in kilos per cubic meter (kg/m^3^), whereas *u* indicates the velocity vector of the fluid, which is measured in meters per second (m/s).

Presented here are the Navier-Stokes equations, which illustrate the concept of conserving momentum:


$$\rho \left(\frac{\partial u}{\partial t}+u\frac{\partial u}{\partial x_{l}}\right)=-\frac{\partial u}{\partial x_{l}}P+\mu {\left(\frac{\partial u}{\partial x_{l}}\right)}^{2u}+\rho g$$ (2)

*P* is the pressure measured in pascals (Pa), *μ* denotes the dynamical viscosity of the medium measured in pascal-seconds (Pa.s), and *g* denotes the acceleration caused by gravity measured in meters per second squared (m/s²).


$$D=\frac{1}{2}\rho V^{2}SC_{d}$$ (3)

The drag force, denoted as *D*, is determined by the velocity, *V*, and the reference area or projected area of the object, denoted as *S*. The drag coefficient, *C*_*d*_, is a dimensionless quantity that quantifies the drag of an object in a fluid.


$$Re=\frac{\rho vL}{\mu }$$ (4)

Here, *ρ* and *μ* indicate the density (kg/m^3^) and viscosity (Pa.s) of water at 25^o^ C, *v* represents the velocity (m/s) of the vehicle, and *L* represents the typical length of the AUV, which is 2.5 meters.

The current situation was designed using the Ansys 2022 R1 program. The simulation was conducted in a steady-state manner, employing the pressure-based solver with a uniform density. The simulation using the *k*-omega SST turbulence design. Initially, the velocity at the entry of the domain remained constant at 0.5 m/s. It was subsequently increased by 0.5 m/s until it reached a velocity of 2 m/s. Simultaneously, the static gauge pressure at the output was adjusted to a value of zero. The no-slip criterion is applied to both the AUV and the walls of the fluid domain.

### 3.3 Numerical methodology

Given that the AUV was simulated in a submerged environment at a short depth, it was assumed that the water density and viscosity were conventional values. The intensity and length scale approach were used to consider variations in water velocity. To be more specific, a turbulence intensity of 6% was applied throughout a distance equivalent to 7% of the distinctive length, or 0.175 m. A second-order discretization approach was employed to solve the Reynolds-Average Navier-Stokes (RANS) equations. An integrated pressure-velocity coupling system was established. Great care was taken to ensure the precision of the numerical values, and the simulation was iterated until it achieved convergence, yielding root mean square residual values of 10^-6^.

#### 3.3.1 Adjoint optimization.

Adjoint optimization is a promising approach for creating and developing aerodynamic designs, such as those for aeroplane wings, vehicle bodywork, or wind turbine blades [50]. The system incorporates the adjoint methodology, optimization methods, and computational fluid dynamics (CFD) principles.

#### 3.3.2 Adjoint equations.

The adjoint approach allows for the efficient calculation of the gradients of an objective function in relation to design components. The objective function of the system is typically associated with optimizing aerodynamic performance, specifically by minimizing drag or maximizing lift. The engineering parameters refer to the components’ characteristics, including the airfoil’s thickness or the wing’s twist, that may be adjusted to determine the shape of the geometry. J. Brezillon and N. R. Gauger provide explicit definitions of the mathematical formulas in their paper [51].


$$R^{P}=-\frac{\partial \bar{v_{i}}}{\partial x_{i}}=0$$ (5)

$$R_{i}^{v}=v_{j}\frac{\partial v_{i}}{\partial x_{j}}+\frac{\partial p}{\partial x_{i}}-\frac{\partial }{\partial x_{j}}\left[\left(\nu +\nu_{t}\right)\left(\frac{\partial v_{i}}{\partial x_{j}}+\frac{\partial v_{j}}{\partial x_{i}}\right)\right]=0\;$$ (6)

$$R_{i}^{z}=Convection+Diffusion+Production+Dissipation=0$$ (7)

In this context, “ $V_{i}$” represents the preliminary velocity, “ *p*” represents the preliminary pressure, and “ *ν*” and “ $\nu_{t}$” represent the turbulent kinematic viscosity. The surface and volume integrals for the objective function *F* are as follows:


$$F=\int_{S}^{0}F_{s}dS+\int_{S}^{0}F_{Ω}dΩ$$ (8)

$$F_{avg}=F+\int_{Ω}^{0}qR^{p}dΩ+\int_{Ω}^{0}u_{i}R_{i}^{v}dΩ$$ (9)

Here, *q* and $u_{i}$ are the adjoint variables; the terms “adjoint pressure” and “adjoint velocity” refer to specific components that are incorporated into the solution process. The sensitivity of the objective function to the movement of the surface elements (design parameters) may be determined by solving the adjoint equations using the provided formula:


$$\frac{\delta F_{avg}}{\delta b}=-\int_{S_{w}}^{0}\left[\left(v+v_{t}\right)\left(\frac{\partial v_{i}}{\partial x_{j}}+\frac{\partial v_{j}}{\partial x_{i}}\right)-qn_{i}\right]\frac{\partial v_{j}}{\partial x_{k}}\frac{\partial x_{k}}{\partial b_{m}}dS$$ (10)

Furthermore, the adjoint far-field boundary requirement states that the geometric position of the far-field remains constant and that the free stream conditions are applied in that region. Adjoint Optimization entails calculating the product of an equation in relation to the relevant parameters and then modifying the parameters to optimize the function. Fig 5 illustrates a schematic illustration of the adjoint technique utilized in the current study. The adjoint approach starts with defining the design variables, here for this research, drag force was observed, and the adjoint method was mainly applied to the wing of the AUV as it is not only one of the major parts for the performance of the AUV but also it will reduce most of the computational time. The Ansys design settings are used to configure factors such as apparent drag, proportion rise, and pressure during the creation of optimization targets. In addition, the adjoint solution technique is employed, utilizing the lowest squares cell-based slope. The pressure was calculated using a 2nd-order approach, which ensured an accurate depiction of the pressure range in the collected data. To get an optimal design, a systematic sequence of operations is employed. First, the fundamental stream formulas are resolved, and then the acquired results are carefully examined using established methods. The chosen variable for observation in this scenario is the drag force, which is quantified as a percentage increase. Modify the geometry as needed in the regions where the increase is implemented. The new geometry is generated by the process of mesh morphing, which involves converting the sensitivity of the surface shape into the sensitivity of the control points. The process entails progressively improving the design solution by altering the mesh, conducting flow simulations using the updated geometry, and analyzing the outcomes. If the outcomes are not optimal, the optimization targets are adjusted, and the process is iterated. This iterative procedure persists until the optimal design solution is attained.

The first step entails choosing a collection of representative photos. More precisely, *N* snapshots are selected from the numerical simulation results at regular time intervals. The snapshots generate a sample information matrix *U*, which integrates both temporal and geographical dimensions, as explained in Eq. (11). Here, *M* is the number of grid nodes. These snapshots are then assembled into a data matrix, $U\left(x_{i},t_{j}\right)$


$$U\left(x_{i},t_{j}\right)\;=\;\left[\begin{array}{ccc} u\left(x_{1},t_{1}\right) & u\left(x_{1},t_{2}\right) & \begin{array}{cc} \cdots & u\left(x_{1},t_{N}\right) \end{array}\\ u\left(x_{2},t_{1}\right) & u\left(x_{2},t_{2}\right) & \begin{array}{cc} \ldots & u\left(x_{2},t_{N}\right) \end{array}\\ \begin{array}{c} \vdots \\ u\left(x_{M},t_{1}\right) \end{array} & \begin{array}{c} \vdots \\ u\left(x_{M},t_{2}\right) \end{array} & \begin{array}{cc} \begin{array}{c} \vdots \\ \ldots \end{array} & \begin{array}{c} \vdots \\ u\left(x_{M},t_{N}\right) \end{array} \end{array} \end{array}\right]$$ (11)

Typically, the spatial dimension *M* is significantly greater than the temporal dimension *N*. Hence, the covariance matrix derived from the collection of sample snapshots *U* is a square matrix with dimensions *M × M*, which poses difficulties in calculating its eigenvalues and eigenvectors. In order to resolve this problem, this study utilizes the Snapshot POD technique to conduct modal decomposition on the matrix *U* [47].

Calculate the average time of each collected sample,


$$\bar{U}\left(x_{i}\right)=\frac{1}{N}\overset{N}{\underset{j=1}{\sum }}U\left(x_{i},t_{j}\right)$$ (12)

The vibrating flow field can be derived by subtracting the temporal average from the first sample picture.


$$\hat{U}\left(x_{i},t_{j}\right)=U(x_{i},t_{j})-\bar{U}\left(x_{i}\right)$$ (13)

The covariance matrix *C*,


$$C=\frac{1}{M}{\hat{U}}^{T}\hat{U}$$ (14)

Next, we calculate the eigenvalues and eigenvectors of matrix *C*.


CA=λA (15)

The eigenvectors of matrix *C* are represented by *A =  (A*_*1*_*, A*_*2*_*,..., A*_*k*_), whereas the eigenvalues of matrix *C* are represented by *λ =  (λ*_*1*_*, λ*_*2*_*,..., λ*_*k*_) ^*T*^ and they were organized in a descending sequence. The POD modes $\phi_{i}$ are formed by utilizing the eigenvectors and the initial fluctuation fields $\hat{U}\left(x_{i},t_{j}\right)$ [47].


$$\phi_{i}\left(x\right)=\frac{1}{\sqrt{\lambda_{\kappa }}}\sum_{i=1}^{N}A_{j}\hat{U}\left(x_{i},t_{j}\right)$$ (16)

These modes are mutually independent and capture the most important characteristics of the flow. The truncated series of the most energetic Proper Orthogonal Decomposition (POD) modes can be used to recreate the preliminary flow field.


$$U\left(x,t_{j}\right)=U\left(x\right)+\sum_{i=1}^{k}a_{i}\left(t_{j}\right)\cdot {\text{Φ}}_{i}\left(x\right)$$ (17)

The steady-state hypothesis simplifies the problem by disregarding temporal change and focusing instead on spatial patterns. This approach facilitates the identification of predominant structures in the flow field and minimizes computational complexity for further analysis or control applications.

### 3.4 Computational efficiency

The computational efficiency of the proposed method was evaluated to assess its practical applicability. The simulations were performed using 8 computational cores with 32 GB of memory on a standard personal computer. The total runtime (wall-clock time) for a single optimization cycle was approximately ± 16200 seconds or 4.5 hours, including the adjoint calculations and Proper Orthogonal Decomposition (POD) post-processing took approximately ± 2450 seconds. Techniques such as parallel processing were employed to enhance computational performance. Proper Orthogonal Decomposition (POD) substantially diminishes the complexity of the computational problem by finding predominant modes in the flow field, hence reducing the resources required for subsequent simulations. Concentrating on a limited number of predominant modes (such as the primary mode that accounts for 96% of the energy) facilitates the reconstruction of critical flow characteristics while minimizing the computational expense associated with modeling the complete turbulence spectrum.

### 3.5 Testing mesh and grid independence

The tetrahedral type of grids with no structure approach is employed to create the mesh. The tetrahedral approach is particularly suitable for complex or irregular structures. The area of computation is divided between two separate areas: the close field and the distant field, as depicted in Fig 6(c). The close field is distinguished by a denser network encircling the body to precisely record the wake zone. The mesh dimensions are indicated in Table 2, and the overall number of components is 2.27 million. Furthermore, boundaries between layers are precisely recorded using inflation layers. Fig 6(a) illustrates the arrangement of the grid in the fluid realm. To ensure an accurate depiction, the y + quantity is determined by calculating the Reynolds number as 12.44 ×  10^5^. The first layer thickness is specified as 0.0240 mm, and the total number of layers is set at 20. A growth rate of 1.2 is employed and the implicated inflation is displayed in Fig 6(b). The overall number of elements was calculated by a grid independence test, as illustrated in Fig 7. The experiment was conducted with seven varying quantities of components, and the force of drag was recorded. It was found that after reaching 2.2 million elements, the drag force remained rather constant. Therefore, 2.27 million number of elements were chosen to reduce computational power as denoted by the optimum grid point in Fig 7.

**Table 2:** A mesh-size database.

Mesh	Element Size
Distant field	230 mm
Close field	35 mm

### 3.5 Model validation

The entire experiment and design were predicated on a study conducted by Olmos et al. [49]. The validation process was conducted in accordance with the findings of this investigation. Fig 8 depicts the validation process, which involves comparing the drag force with the velocity. Compared to the investigation conducted by Olmos et al. [49], the present study demonstrates a relatively lower level of drag. However, the discrepancy among the four comparisons at a velocity of 1 m/s is minimal.

## 4. Results and discussions

### 4.1 Optimization of design

**Fig 11:**
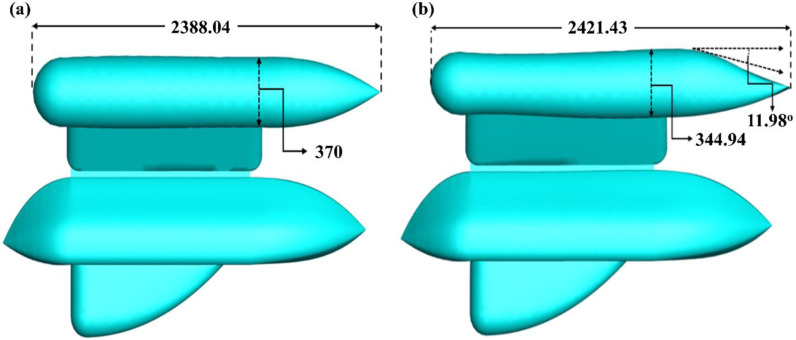
Comparative Analysis of (a) Preliminary and (b) Optimized design (all dimensions are in mm).

Initially, CFD analysis was performed on the design of the AUV. The simulation was conducted under fully submerged conditions, with varying velocities, like the work conducted by Olmos et al. [49]. The initial simulation was performed at a speed of 1 m/s, as it roughly corresponded to the value shown in the validation Fig 8. Then, the adjoint simulation was done for that velocity. Where the drag force of 98.913 N for a 3% change of the geometry got drag force reduced to 95.209 N, about a 4% reduction from the initial drag force. Table 3 and Fig 9 show the drag force reduction up to 9% change of the wing of the AUV. It is observed that 9% is optimal for drag reduction if any further changes the body too much, causing drags to increase rather than decrease. Fig 10 illustrates a gradual and substantial alteration in the trailing edge of the wing. Based on the information provided in Fig 11, the height was reduced from 370 mm to 344.94 mm. Additionally, there is a curvature of 11.98° on the upper side of the wing. The most significant alteration is the increase in the wing’s overall length, which grew from 2388.04 mm to 2421.43 mm.

**Table 3:** Wing percentage changes on drag force.

Change (Percentage)	Drag Force (N)
0%	98.9103
–3%	95.2091
–5%	92.7532
–7%	90.9740
–9%	90.1733

**Fig 12:**
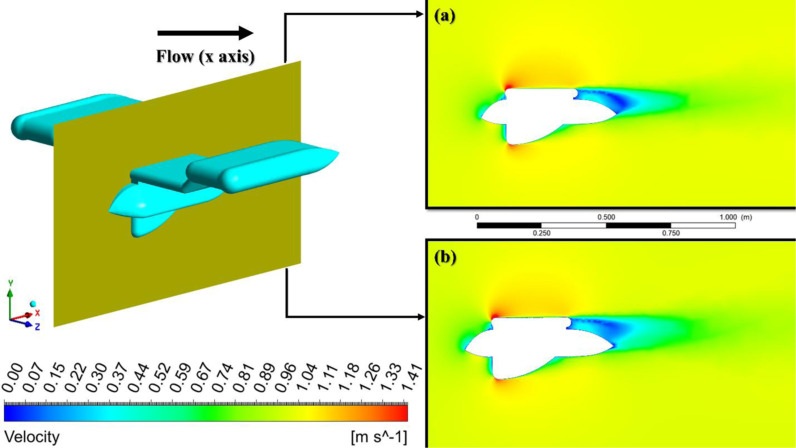
Comparison of the velocity, a) Preliminary, b) Optimized design.

Fig 12 shows the velocity contours for a velocity of 1 m/s on the AUV’s symmetry plane. First and foremost, the dimensions and locations of the wake are of utmost importance. Fig 12(a) exhibits a wider and more extensive wake compared to Fig 12(b). In both designs, red areas can be observed on the upper and lower surfaces of the AUV, particularly near the leading edge. These red regions imply high velocity. The optimized design, as shown in Fig 12(b), has bigger regions of decreased velocity near the body. This indicates a greater presence of turbulent activity and increased dissipation of energy, both of which lead to an elevation in drag. Furthermore, as deduced from the velocity contours, the pressure gradient and boundary layer behavior also exhibit disparities between the two. A diminished wake signifies that the separation of airflow from the outer surface of the object occurs at a later point, typically resulting in a reduction in drag [52]. In addition, the flow attachment in Fig 12(b) is more prominent, as indicated by the velocity contours. These contours show that the air remains attached to the surface of the body for a greater distance, particularly in the blue region at the tip of the trailing edge. Effective airflow control minimizes the creation of turbulent wake areas, as evidenced by the size of the light blue region, resulting in a reduction in drag. In Fig 12(b), the flow distribution across the body, especially along the trailing edge, exhibits a more uniform high-velocity pattern. This indicates efficient flow control and reduced energy loss to turbulence. Fig 12(b) experiences a more advantageous pressure gradient, resulting in a delay of boundary layer separation and a reduction in the low-pressure wake behind the object. This, in turn, helps to decrease drag [53–55].

**Fig 13:**
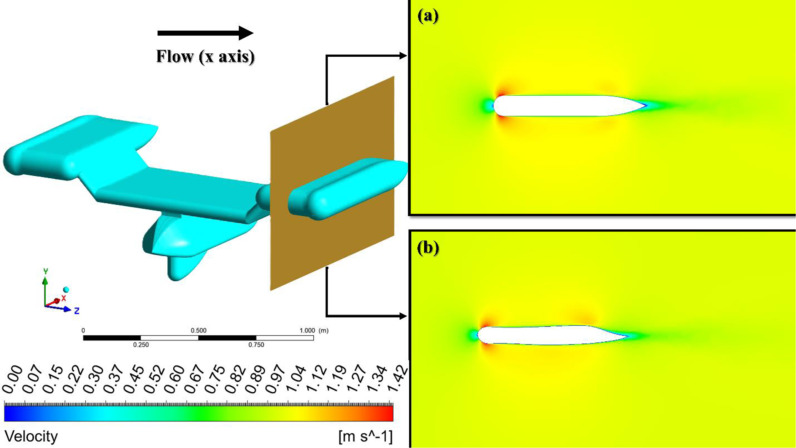
Comparison of the velocity contour wing, (a) Preliminary, (b) Optimized design.

Fig 13 shows the velocity contours for the optimized geometry, specifically the wing of the AUV. Similar to Fig 12, there are red areas present on both the upper and lower surfaces of the wing toward the front edge. The basic design, seen in Fig 13(a), exhibits a broader wake in comparison to the optimized design shown in Fig 13(b). This is attributed to the streamlined velocity flow resulting from the reduction in surface area on the top wing surface at the trailing edge, leading to decreased pressure and higher velocity. This results in enhanced laminar flow and decreased wake turbulence produced downstream of the trailing edge.

**Fig 14:**
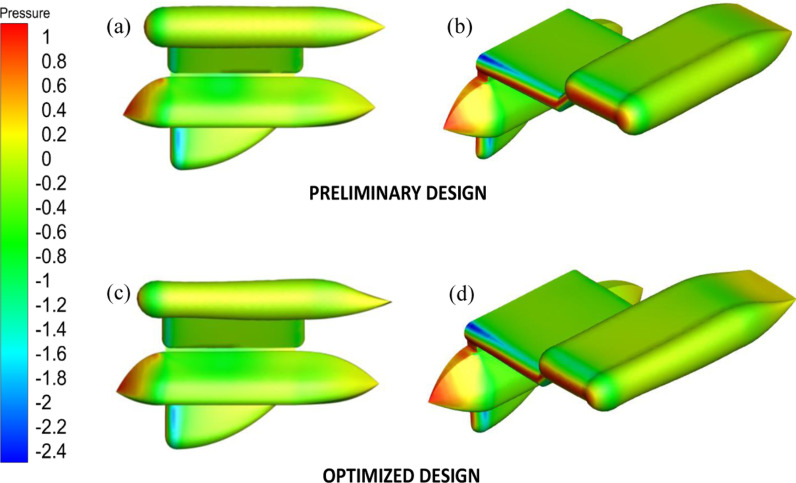
Pressure contour for Preliminary and Optimized design.

Fig 14 displays the pressure distribution for both the preliminary and optimized designs. In both the preliminary and optimized designs (Fig 14(a) and Fig 14(c)), the side view shows a similar pressure distribution. The front of the designs experiences excessive pressure, as it is the stagnation point. As the pressure goes from the leading edge to the trailing edge, it gradually diminishes. Nevertheless, the preliminary distinction between the designs is most evident at the rear edge of the wing, where the adjoint technique saw the most significant alteration. By decreasing the surface area of the upper wing surface, the pressure was increased, as shown in Fig 14(d), leading to an increase in velocity and resulting in a more streamlined fluid flow and disturbance in the wake [56,57].

**Fig 15:**
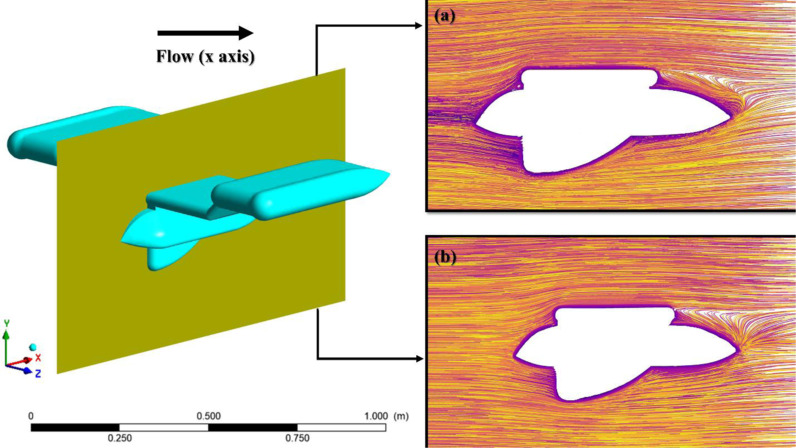
Streamline flow visualization of (a) Preliminary design; (b) Optimized design.

The streamlines depicted in Fig 15 can be utilized to assess the aerodynamic efficiency of the AUV. The streamlines in the Preliminary design (Fig 15(a)) exhibit turbulent flow, resulting in recirculation or reattachment and the formation of vortex pairs that are visible on the upper surface of the leading edge of the AUV. Nevertheless, the most notable addition can be observed at the back end of the preliminary design, where the optimized design shown in Fig 15(b) demonstrates a greater level of smoothness and closely adheres to the body, particularly in crucial regions such as the leading edges and trailing end. This adherence indicates that the flow remains attached to the surface of the body for a longer period of time, resulting in a reduction in flow separation and a delay in reattachment. This results in a decrease in wake generation and an overall reduction in drag [58,59].

### 4.2 POD analysis results

**Fig 16:**
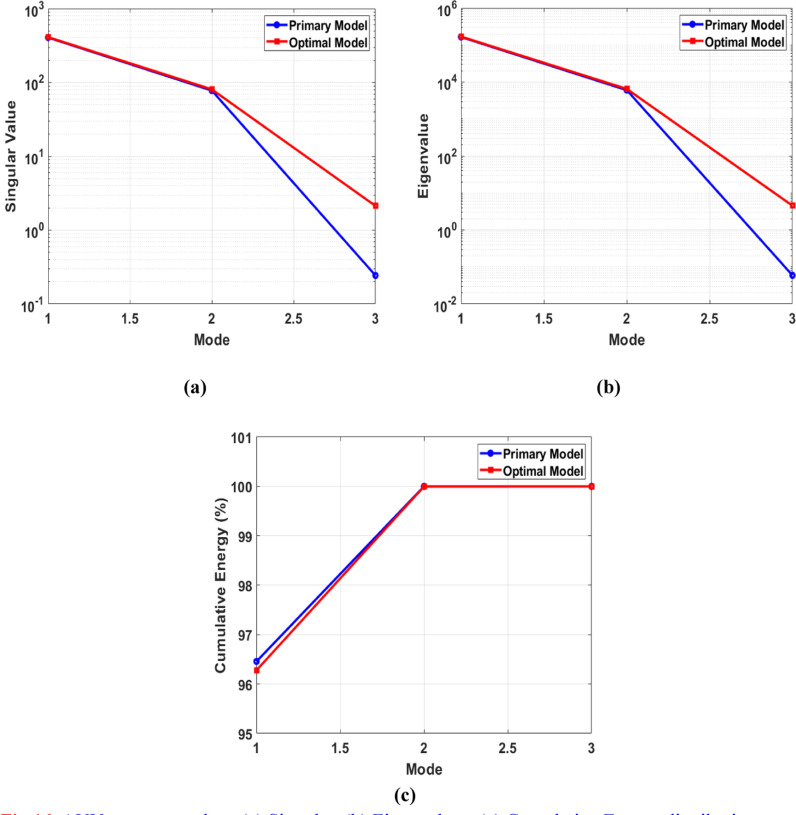
AUV symmetry plane (a) Singular, (b) Eigenvalues, (c) Cumulative Energy distribution.

Fig 16 illustrates the outcomes of a Proper Orthogonal Decomposition (POD) study conducted on the AUV’s symmetry plane at a velocity of 1 m/s. The “Preliminary Design” refers to the outcomes obtained without utilizing the adjoint method, whereas the “Optimal Design” refers to outcomes obtained after applying the adjoint method. Fig 16(a) shows the singular values associated with each index or mode derived by performing singular value decomposition (SVD) on the data matrix. Singular values represent the importance or relevance of each mode. The larger single values correspond to the modes or indices that have a greater impact on the reconstruction of the preliminary data. In Fig 16(a), the preliminary design shows a significant decline in the singular values, starting from around 10^3^ for the first mode. The quick decrease indicates that the first mode mostly influences the flow field of the preliminary design, while the subsequent modes have a substantially smaller impact. This level of dominance suggests ineffective flow control, where elevated amounts of turbulent kinetic energy contribute to heightened skin friction drag. In the improved design, the singular values exhibit a slower rate of reduction. The initial mode commences at a comparable value of 10^3^, however, the decrease is less pronounced. This suggests that the enhanced form results in a fairer allocation of flow characteristics among the different modes. The decline in predominance of the initial mode indicates a decrease in the intensity of large turbulent structures, leading to a decrease in skin friction drag and an enhancement in the overall flow stability and efficiency. Fig 16(b) illustrates the correlation between eigenvalues and index in the second plot. The eigenvalues are associated with the covariance matrix of the dataset and provide crucial information about the amount of energy captured by each mode. Higher eigenvalues are associated with modes that capture a larger amount of energy in the system. In Fig 16(b), the preliminary design demonstrates a substantial reduction in eigenvalues from the first mode (about 10^6^) to the third mode. The sharp decrease in value suggests that most of the energy in the flow field is focused on a small number of modes. This implies the presence of prominent large-scale swirling patterns and areas where the flow separates, leading to substantial resistance forces. Conversely, in the ideal design, the eigenvalues likewise decline, albeit at a slower pace than in the preliminary design. The initial mode starts with a value of 10^6^ which is comparable, but the subsequent modes have higher eigenvalues.

The gradual reduction observed indicates that the enhanced shape efficiently disperses the energy of the flow in a more consistent manner throughout the various modes. A uniform distribution of flow reduces the prominence of large-scale vortices and flow separations, leading to a smoother and more efficient flow around the AUV. Consequently, the reduction of both forms drags, and pressure drag occurs. Now, let’s demonstrate the amount of energy that each mode has captured. Fig 16(c) displays the preliminary design and optimal design in the first mode, both of which capture around 96% of the energy. The preliminary design has slightly more energy than the optimal design. As the mode increases, both designs exhibit an identical increase in energy, ultimately reaching 100%. This means that the majority of the kinetic energy is concentrated in the first few modes, indicating that large-scale structures primarily influence the flow dynamics. However, the optimal design has a more refined flow structure compared to the other design, as demonstrated by the reconstructed velocity contours for each mode in Figs 18 and 19.

**Fig 17:**
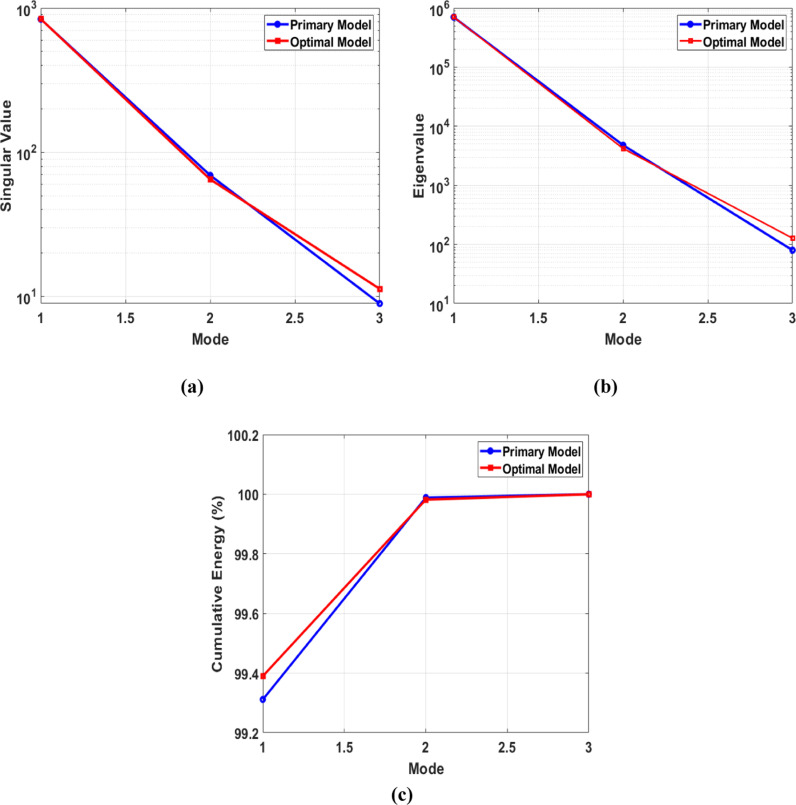
AUV wing plane (a) Singular, (b) Eigenvalues, (c) Cumulative Energy distribution.

Fig 17 displays POD plots for the AUV wing plane, which underwent the preliminary form optimization process. In the singular value plot (Fig 17(a)), it is evident that both designs show a significant decline in singular values from around 10^3^ for the first mode. In the third mode, the optimal design has a higher value than the preliminary design. When comparing the AUV symmetry plane to the wing plane, it is found that the wing plane has a higher singular value. This indicates that the wing geometry retains more energy and is more complex, containing more important features. Similarly, in the case of Fig 17(b), the eigenvalues for the wing plane exceed those of the AUV symmetry plane. In Fig 17(c), it can be noted that the wing plane preserves a higher amount of cumulative energy compared to the symmetry plane. In the first mode, both designs start from a range over 99.2%, while in the symmetry plane, it starts from above 96%.

**Fig 18:**
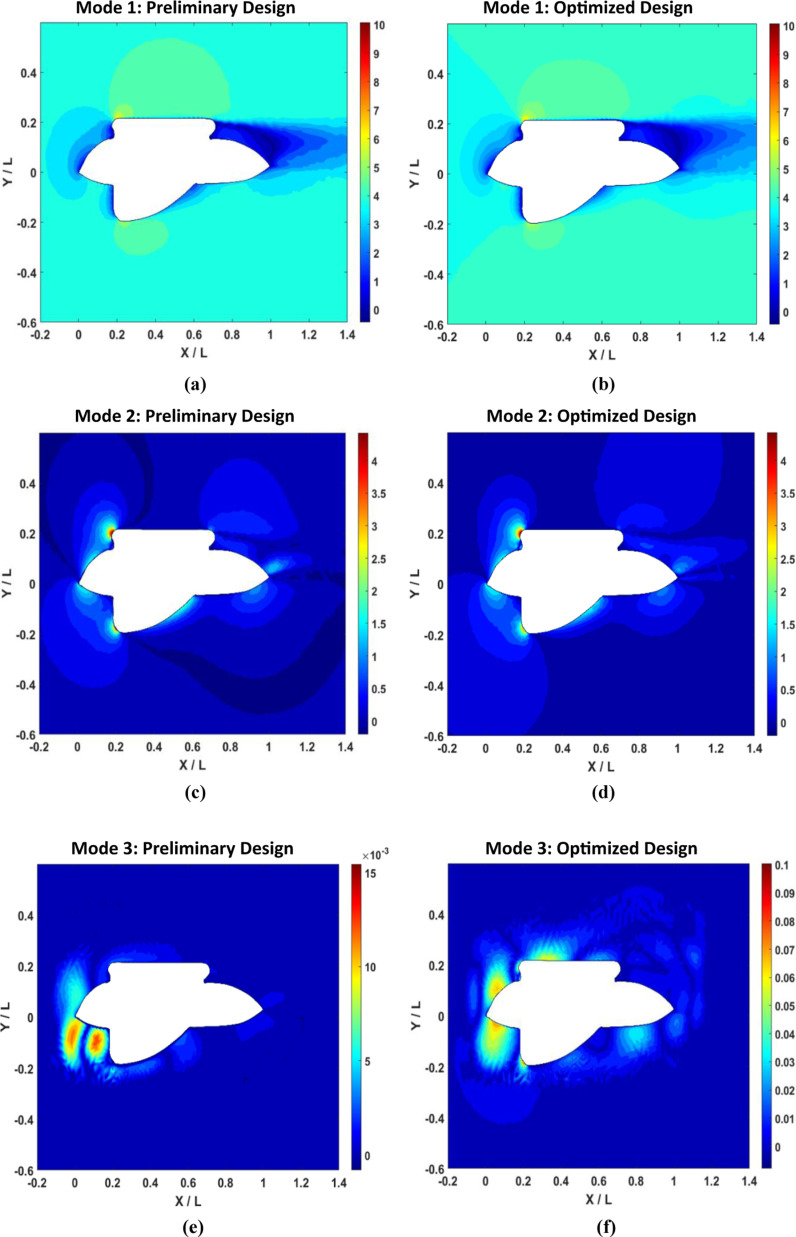
AUV symmetry plane POD-based reconstructed velocity contours.

Now, POD is used to reconstruct velocity contours in different modes, as illustrated in Fig 18. It highlights the main flow features, such as vortices and shear layers, while ignoring less significant details and noise. Fig 18(a) and Fig 18(b) show velocity contours mode 1 for preliminary and optimized design, respectively. In Fig 18(a), high-velocity regions are prominent at the leading edge and along the sides of the AUV, as shown by red and yellow colors. These regions indicate where the fluid undergoes significant acceleration due to the AUV’s geometry, resulting in high dynamic pressure. The sharp velocity gradients at these locations contribute to considerable form drag, a preliminary component of the total drag force experienced by the AUV. The blue regions, indicative of low velocities, suggest areas of flow separation and recirculation zones. These adverse pressure gradients and flow separation phenomena increase pressure drag, further reducing the hydrodynamic efficiency of the AUV. On the other hand, in Fig 18(b), the optimized design first mode shows a more homogenized distribution of high-velocity regions around the AUV. The high-velocity zones are less intense and more smoothly transitioned, indicating a more favorable pressure distribution and lower form drag. The streamlined shape achieved through adjoint optimization reduces the sharp velocity gradients, thereby minimizing adverse pressure gradients. Consequently, flow separation is diminished, as evidenced by the reduced presence of low-velocity blue regions. This leads to a significant reduction in pressure drag and an overall decrease in the total drag force acting on the AUV, enhancing its hydrodynamic efficiency.

For preliminary design mode 2 in Fig 18(c) near the leading edges and the wake of the AUV. High vorticity indicates strong rotational motion in the fluid, typically resulting from flow separation and shear layers forming due to the adverse pressure gradients. These vortical structures are associated with increased turbulence and energy dissipation. The presence of strong vortices suggests significant induced drag, contributing to higher total drag on the AUV and impeding its performance in underwater operations. The optimized design mode 2 (Fig 18(d)) reveals a marked reduction in vorticity around the AUV. The high-vorticity regions are less intense and more diffused, indicating that the optimized shape promotes flow attachment and reduces the formation of shear layers. The smoother surface geometry achieved through adjoint optimization minimizes flow separation and delays boundary layer transition. This reduction in vorticity corresponds to lower induced drag and less turbulent kinetic energy dissipation, enhancing the AUV’s hydrodynamic performance and stability in the fluid environment.

For the final mode illustrated in both Fig 18(e) and Fig 18(f), the POD reconstructed velocity contours showcase the most dominant flow structures containing the most energy while reducing unnecessary flow structures. In Fig 18(e), high velocity is concentrated near the front and sides of the AUV. In those areas, high velocity means there is a lot of energy, specifically turbulent kinetic energy. This diffused pattern suggests a chaotic flow regime, leading to increased energy losses and higher drag forces. The turbulence exacerbates the inefficiency of the flow around the AUV. On the other hand, for the optimized design in Fig 18(f), there is a substantial reduction in the turbulent kinetic energy. The high-turbulence regions near the front and sides of the AUV have diminished, indicating a transition to a more orderly flow regime. This reduction in turbulence correlates with decreased energy losses and drag, significantly improving the AUV’s hydrodynamic performance.

**Fig 19:**
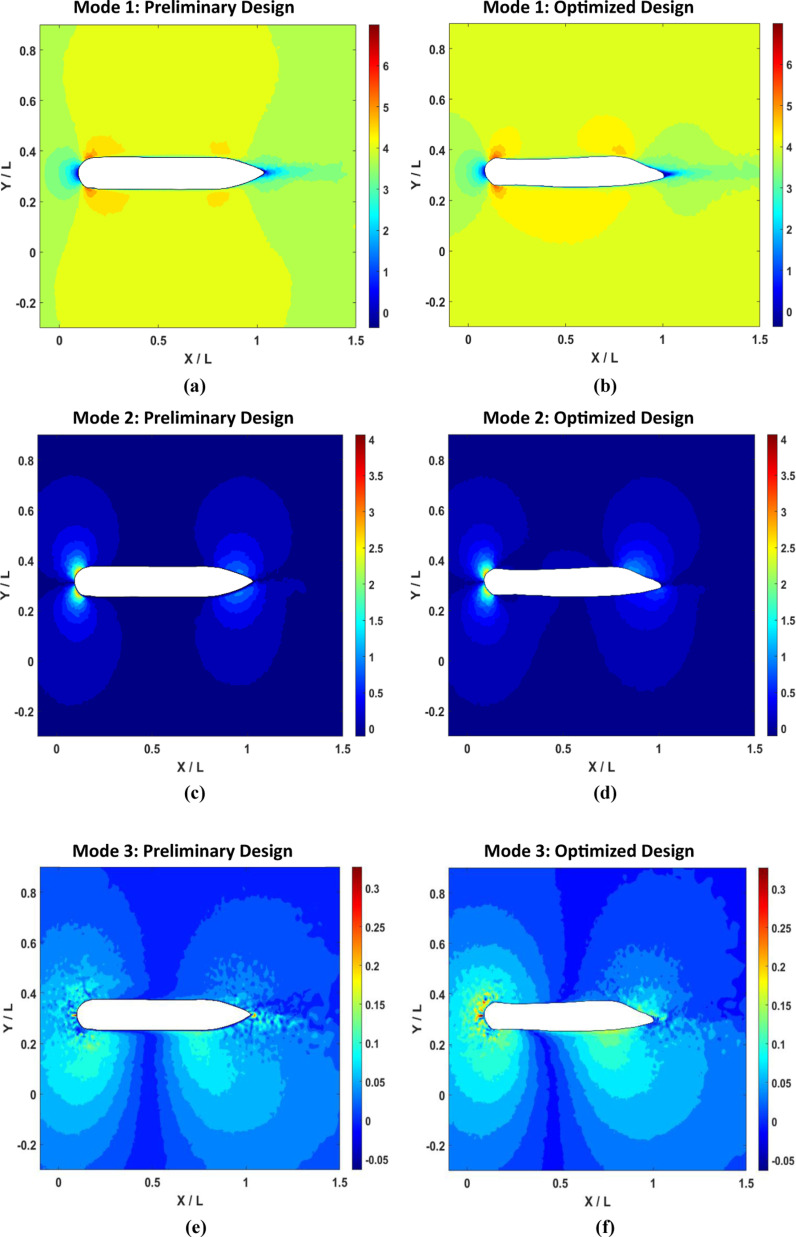
AUV Wing plane POD-based reconstructed velocity contours.

For the wing plane, POD reconstructed velocity contours are given in Fig 19. Whereas, in Fig 19(a), the first mode for preliminary design is high energy regions around the wing, particularly at the leading edge. This indicates substantial flow separation and vortex formation, contributing significantly to drag forces. The wake region behind the wing is extensive and turbulent, suggesting inefficient flow dynamics and increased pressure drag. For the optimized design (Fig 19 (b)), a more streamlined flow pattern can be seen. The high-energy regions are considerably reduced and more evenly distributed along the wing surface, which minimizes flow separation at the trailing edge. Consequently, the wake region is narrower and less turbulent, indicating a significant decrease in form drag and improved hydrodynamic efficiency. In the second mode for both preliminary (Fig 19(c)) and optimized design (Fig 19(d)), there can be seen velocity changes in the leading and trailing edges of the wing, and those velocity changes can represent vortex structures. In both designs, high velocity is at the leading edge or stagnation point; however, in the optimized design, there is a gradual decrease, whereas in the preliminary design, it retained more velocity. Also, it can be seen in the trailing edge. In addition to decreasing velocity value, it removes a vortex bubble in the lower surface of the trailing edge of the wing. Making the upper surface into a curve reduces the surface area, causing pressure drop and increasing the velocity, ultimately reducing drag force. The third POD mode before optimization (Fig 19(e)) highlights smaller eddies and recirculation zones, indicating areas of significant viscous drag. High-velocity gradients in the wake region further emphasize regions of high viscous shear stress and energy dissipation, contributing to overall drag. After optimization in Fig 19(f), there is a considerable reduction in turbulence and improved flow separation control, and it is more prominently visible in the trailing edge, as mentioned before, due to the curve feature in the upper surface, the fluid can flow more smoothly.

Following the analysis of a wing’s performance at a velocity of 1 m/s, the same wing underwent testing at various velocities, as indicated in Table 4, after a 9% modification to its body. The data shown in Table 4 illustrates the impact of aerodynamic optimization on the decrease in drag force at various velocities for a wing design. The wing, which has been improved, is moving at a velocity of 0.5 meters per second. It shows a decrease in drag of 16%, going from 25.05892 N to 21.130592 N. The pace of decrease remains consistent at 1 meter per second, leading to a 9% drop. When the speed is 1.5 m/s, the drag force decreases by 11%. Notably, the most substantial decrease is when the velocity is 2 m/s, leading to a 17% decrease in drag.

**Table 4:** The data of preliminary wing and Optimized wing drag force at various velocity.

Velocity (m/s)	Drag Force (N)		Reduction (%)
	Preliminary Wing	Optimized Wing	
0.5	25.05892	21.130592	16%
1	98.910316	90.173366	9%
1.5	214.27133	190.58602	11%
2	386.34196	320.89933	17%

### 4.3 Power efficiency

**Fig 20:**
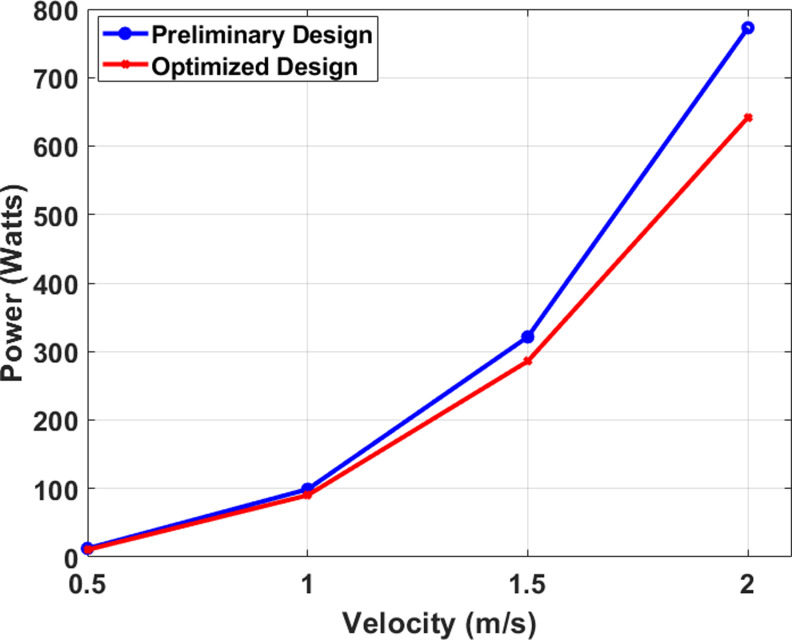
Comparison of Power consumption for Preliminary and Optimized design.

The drag force encountered by a moving vehicle is a crucial factor in its total power consumption, as a higher drag force requires more energy to overcome fluid resistance. Therefore, a decrease in drag can significantly contribute to improved power efficiency. Fig 20 illustrates that both configurations exhibit minimal power usage at low velocity. This is because the power is exactly proportional to the drag force and velocity. As velocity rises, the power usage also increases. In addition, the design must effectively counteract the drag force exerted by the fluid as it propels ahead, necessitating a significant amount of power to overcome this resistance. Comparing the two designs, it is evident that the preliminary design exhibits higher power consumption. At its maximum velocity of 2 m/s, the preliminary design consumes around 790 watts of power, while the optimized design consumes 630 watts, indicating an approximately 20.25% improvement. As the velocity increases, the power consumption of the optimized design increases due to the decrease in drag force and the resulting improved fluid flow.

## 5. Conclusions

This study significantly enhances the field of naval engineering by providing useful insights into methods for reducing drag in underwater vehicles. This research demonstrates the significant potential for optimizing fluid dynamics in an underwater setting using the adjoint approach. Applying theoretical optimization frameworks to build a winged AUV demonstrates how Computational Fluid Dynamics may efficiently improve hydrodynamic efficiency and operational performance. The main findings are listed below:

- The study observed decreases in drag force at different velocities by employing adjoint-based optimization strategies. More precisely, the drag reductions varied from 9% when the speed was 1 m/s to a significant 17% when the speed was 2 m/s. This shows that technology can efficiently control dynamic pressures and improve flow separation at greater speeds.
- The Proper orthogonal decomposition (POD) analysis identified the main flow configurations that caused an increase in drag force in the AUV. The primary advantage was that the majority of the kinetic energy, almost 100%, was concentrated in the lower surface of the preliminary design’s leading edge. The optimized design effectively distributed this energy evenly along the leading edge. Furthermore, the wing plane also noted a reduction in wake development behind the AUV’s wing as it balanced the energy.
- The analysis of power efficiency has demonstrated that the optimized design effectively reduces both drag and power consumption. As the speed increases, the power efficiency also increases. The maximum power efficiency was seen at 630 watts with a velocity of 2 m/s, achieving a power efficiency of 20.25%.
- This research demonstrates that employing the adjoint approach alone on a certain geometry section can yield highly successful results.
- Advanced computational techniques, such as CFD and adjoint improvement methodologies, have been instrumental in bridging the gap between theoretical optimization frameworks and practical engineering solutions. These technologies facilitated accurate alterations in the wing design to attain specific enhancements in performance.

As with every project, this study has its limitations. The following suggestions are proposed to improve the outcomes of this work:

- Conduct experimental water tunnel tests to analyze how the AUV behaves under actual conditions. Compare the experimental results with numerical simulations, including detailed contours.
- Apply advanced machine learning methods, such as Dynamic Mode Decomposition (DMD) and Physics-Informed Neural Networks (PINNs), to better capture complex flow dynamics and enhance the accuracy of optimization processes.

These methods can provide deeper insights into fluid-structure interactions and improve the robustness of the numerical models. Ultimately, this study provides significant knowledge in the field of naval engineering, specifically on the development and enhancement of submersible vehicles. This paves the door for future improvements in the efficiency and performance of hydrodynamics, using computer optimization to create environmentally friendly and operationally efficient designs.
